# A General Synthesis of C8-Arylpurine Phosphoramidites

**DOI:** 10.3390/molecules14093339

**Published:** 2009-09-02

**Authors:** Vorasit Vongsutilers, Jonathan R. Daft, Kevin H. Shaughnessy, Peter M. Gannett

**Affiliations:** 1 West Virginia University, Department of Basic Pharmaceutical Sciences, P.O. Box 9530, Morgantown, WV 26506, USA; E-mails: vorasit.v@chula.ac.th (V.V.); jdaft@mail.rx.uga.edu (J.R.D.); 2 Department of Chemistry and Center for Green Manufacturing, The University of Alabama, Box 870336, Tuscaloosa, Alabama 35487-0336, USA; E-mail: kshaughn@bama.ua.edu (K.H.S.)

**Keywords:** C8-arylpurine synthesis, Suzuki coupling, phosphoramidite

## Abstract

A general scheme for the synthesis of C8-arylpurine phosphoramidites has been developed. C8-Arylation of C8-bromo-2′-deoxyguanosine is the key step and has been achieved through the use of a Suzuki coupling. Since the coupling reaction is conducted under aqueous conditions, it is unnecessary to protect and then deprotect the hydroxyl groups, thus saving several steps and improving overall yields. Once the C8-arylgroup is introduced, the glycosidic bond becomes very sensitive to acid catalyzed cleavage. Protection of the amino groups as the corresponding *N,N*-dimethylformamidine derivative improves stability of the derivatives. Synthetic C8-arylpurines were successfully used to prepare synthetic oligonucleotides.

## Introduction

C8-arylpurines are DNA adducts known to form during exposure to carcinogenic arylhydrazines [1,2] and related compounds [3]. These DNA adducts may be related to arylhydrazine carcinogenesis, whose mechanism is unknown, but may be related to either mutagenic or conformational effects of the modified purines. Recently, it has been proposed that the mutagenic or carcinogenic nature of the C8-arylpurine adducts is related to the effect they have on DNA conformation. In particular, C8-arylpurine adducts tend to shift the normally preferred B DNA conformation to the Z form. Therefore, it is of interest to prepare oligonucleotides that contain this particular type of DNA adduct. 

In order to prepare C8-arylpurine modified oligonucleotides so that conformational and mutational effects can be probed, it is necessary to develop procedures whereby C8-arylpurine modified oligonucleotides can be made and this, in turn, requires the synthesis of the corresponding phosphoramidites. Unfortunately, the reported procedures used to synthesize C8-aryl-2'-deoxy-guanosine DNA adducts give poor yields and lack scope and reproducibility [4]. Thus, we report here a new procedure for the synthesis of C8-arylpurine phosphoramidites.

In selecting an appropriate arylation reaction, a number of considerations were made. First, unprotected nucleosides have poor solubility in organic solvents and it would be advantageous to use a reaction that was amenable to aqueous conditions. Second the reaction needs to be efficient at generating a carbon-carbon bond between unsaturated substrates. Third, the reaction must have broad scope for the synthesis of a variety of C8-arylpurines so that a wide range of these DNA arylated adducts can be studied. A reaction that meets all three of these conditions is the Suzuki coupling reaction since C8-halogenated purines are readily prepared [5,6,7,8] and water soluble palladium ligands for this reaction are now available [9,10]. Finally, the Suzuki coupling reaction is typically very efficient at coupling unsaturated substrates, so it is an excellent choice for synthesizing C8-arylpurines. Finally, many arylboronic acids are commercially available or are easily prepared, meaning a wide variety of C8-arylpurines may be synthesized via the Suzuki coupling reaction.

## Results and Discussion

**Scheme 1:**
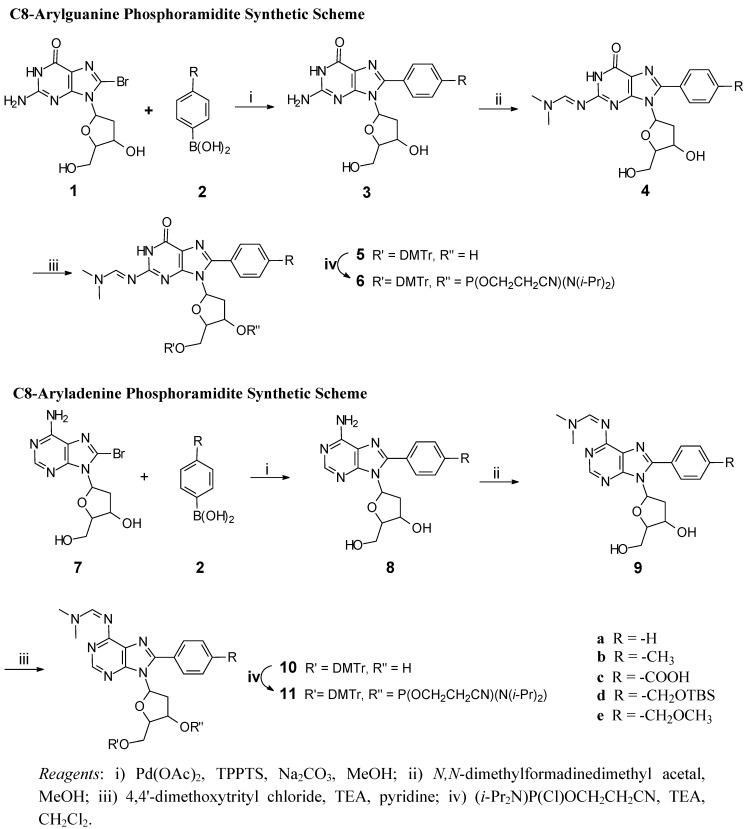
General Synthetic Route to C8-Arylpurine Phosphoramidites.

The synthetic schemes employed for the synthesis of C8-arylpurines phosphoramidites are shown in Scheme 1. In either case, the first step utilizes the Suzuki coupling reaction between the C8-bromopurine **1** or **7** and an aryl boronic acid **2** to give the corresponding C8-arylpurine **3** or **8**. This cross-coupling reaction was conducted in aqueous methanol containing sodium carbonate and palladium acetate, at 80 °C for the C8-arylguanine products or room temperature for the C8-aryladenine products. TPPTS was used as the catalyst in all cases. The reactions were readily followed by TLC and run until the C8-bromopurine starting material had been consumed. The isolation of the product typically required only adjusting the pH to approximately 7, as this caused the precipitation of the cross-coupling product, which was then isolated by filtration. The product was often sufficiently pure following a methanol wash, such that further purification was unnecessary. Yields were typically 80% or better for the phenyl and tolyl derivatives and 67-80% for the carboxyl and TBS protected hydroxymethyl derivatives. 

The subsequent steps for phosphoramidite synthesis are typical. First, the purine amino group was protected as the *N,N*-dimethylformamidine derivative (**4** and **9**) by simply stirring the cross-coupled product in *N,N*-dimethylformamidine dimethyl acetal and methanol. The reactions were essentially quantitative and isolation only required partial removal of solvents, water dilution, and filtration of the precipitated product. The *N,N*-dimethylformamidine group has been used as a protecting group for either guanine or adenine based phosphoramidites in place of isobutyl (guanine bases) or benzoyl (adenine bases). In the course of this work we found that the glycosidic bond of the C8-arylguanines was very labile when the exocyclic amino group was protected with the normal isobutyryl group (guanine) or benzoyl group (adenine). Dialkylformamidine protection of exocyclic amino groups decreased the lability of the glycosidic bond rendered the respective nucleosides less prone to decomposition during phosphoramidite synthesis [11,12]. 

This increased stability facilitates phosphoramidite synthesis, simplified purification and gave higher yields. Still, even with this modification, chromatographic purification on silica gel caused near complete decomposition, so chromatography was conducted on basic alumina. Nevertheless, a significant amount of material was lost due to decomposition, especially for the C8-arylguanine intermediates. Since the ‘crude’ products were usually sufficiently pure (95% or better by NMR), they were routinely used without chromatographic purification and chromatographic purification was only used for preparing analytical samples.

The final two steps for phosphoramidite synthesis are the protection of the 3'- and 5'-sugar hydroxyl groups. The 5’-hydroxyl group is protected as the dimethoxytrityl ether (DMTr) by reaction of either **4** or **9** with DMTr-Cl in pyridine containing triethylamine (TEA). As described above, the C8-arylguanine derivatives are susceptible to cleavage at the glycosidic bond. In addition, the DMTr-ether is likewise very prone to acid cleavage. The DMTr ethers did require purification and this was achieved by chromatography on alumina but with loss of product due to some decomposition. Chromatography on silica resulted in complete decomposition of the DMTr products.

Phosphotidylation completed phosphoramidite synthesis and this material was typically used with minimal purification. In particular, after removal of the reaction solvent and excess TEA, the TEA hydrogen chloride salts were removed by dissolving the reaction mixture in a mixture of benzene and tetrahydrofuran (THF) and then filtration to remove the TEA salts. Any residual moisture was removed by several co-evaporations with benzene and then drying *in vacuo* over phosphorus pentoxide. The resulting phosphoramidites were stable at room temperature or below, as long as moisture was excluded. Chromatographic purification was possible and used to obtain analytical samples but at the cost of substantial loss of the products **6** or **11**.

**Scheme 2:**
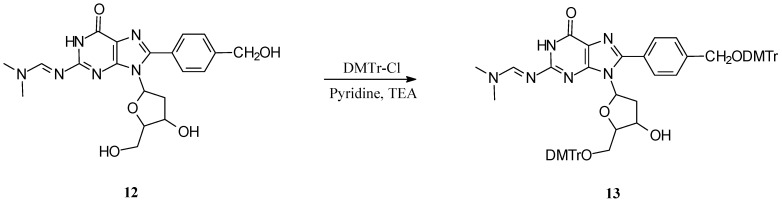
Formation of the bis-DMTr ether of C8-(4-hydroxymethyl)phenyl-2′-deoxyguanosine during protection of the 5′-OH group.

These phosphoramidites have been successfully used to make C8-arylpurine modified oligonucleotides, including ^5'^CGCGCG*CGCG^3'^, ^5'^TGTGTG*TGTG^3'^, and ^5'^CACACA*CACA^3'^ (G* = 8-phenylguanine, 8-tolylguanine, 8-carboxyphenylguanine, 8-hydroxymethylphenylguanine, and C8-methoxymethylphenylguanine; A* = 8-phenyladenine, 8-tolyladenine). However, the guanine based phosphoramidites formed gels shortly after dissolving in acetonitrile and were manually coupled as they could be immediately used. Interestingly, the free carboxylic acid group of **6c** did not interfere with the oligonucleotide synthesis and no effort was made to protect this group for phosphoramidite synthesis. However, the hydroxyl group in hydroxymethylphenyl derivative **12** did require protection (*i.e.,* compound **6d**) as it otherwise reacts with DMTr-Cl (to give the bis-protected DMTr ether, Scheme 2). In turn, the ether was likely cleaved when treated with TCA during oligonucleotide synthesis, providing two potential sites for coupling. To ensure this did not occur, the hydroxyl methylphenylboronic acid was protected as the TBS ether. This protecting group was stable to all of the reaction conditions used for phosphoramidite synthesis and was deprotected, following oligonucleotide synthesis by treatment with concentrated ammonium hydroxide, which also removed all other protecting groups.

## Experimental

### General

2'-Deoxyguanosine was purchased from USB (Cleveland, OH, USA), tris(3-sulfonatophenyl)-phosphine hydrate sodium salt (TPPTS) was purchased from Strem Chemicals (Newburyport, MA, USA), and all other reagents were purchased from Aldrich Chemical Co (Milwaukee, WI, USA) and used as received. Solvents were purchased from Fisher Scientific (Pittsburgh, PA, USA) and were purified and dried using standard techniques. IR spectra were obtained on a Perkin-Elmer infrared spectrophotometer model 782 in potassium bromide matrix. UV spectra were obtained on a Beckman DU640 spectrophotometer. NMR spectra were obtained on a Varian Inova 600 MHz spectrometer, referenced to TMS. Proton resonances were assigned based on COSY correlations, carbons bonded to protons were assigned by Hetcor correlation, and carbons without protons were assigned on the basis of chemical shift. In the case of multiple isomers the reported chemical shifts refer to the major isomer. Mass spectra (MS) were recorded on a Finnigan LCQ Deca using electrospray ionization. 

### General Procedure for the Suzuki Coupling Reaction

Pd(OAc)_2_ (11 mg, 0.05 mmol), tris(3-sulfonatophenyl)phosphine trisodium (TPPTS; 76 mg, 0.13 mmol), Na_2_CO_3_ (400 mg, 3.77 mmol), compound **1** (0.657 g, 1.90 mmol) and aqueous acetonitrile (20 mL, 33%, pre-sparged with nitrogen) were heated at 80 °C under nitrogen. The arylboronic acid (**2**, 2.35 mmol) was then added and the reaction heated at 80 °C, with stirring, for 2.5 hours (ratio Pd(OAc)_2_:TPPTS:Na_2_CO_3_:**1**:**2** = 1:2.6:75:38:47). The reaction mixture was cooled to room temperature, water (40 mL) added, the pH adjusted to 7 with 10% KH_2_PO_4_ or HCl, cooled in an ice bath, and filtered. The filtercake was dried *in vacuo* to yield the arylated product.

*C8-Phenyl-2'-deoxyguanosine* (**3a**): Yield 83%; IR (KBr) cm^-1 ^3600-3000, 2930, 1740-1555, 1360, 1100; ^1^H-NMR (DMSO-d_6_) δ ppm 10.80 (1H, bs, NH), 7.65 (2H, dd, *J* = 1.8, 7.3 Hz, ArH-3,5), 7.52 (3H, m, ArH-2,4,6), 6.42 (2H, bs, NH_2_), 6.07 (1H, dd, *J* = 6.6, 7.8 Hz, H-1'), 5.14 (1H, d, *J* = 2.4 Hz, 3'-OH), 4.99 (1H, bs, 5'-OH), 4.33 (1H, m, H-3'), 3.79 (1H, m, H-4'), 3.65 (1H, m, H-5"), 3.54 (1H, m, H-5'), 3.17 (1H, p, *J* = 6.9 Hz, H-2"), 2.02 (1H, ddd, *J* = 2.4, 6.6, 13.2 Hz, H-2'); ^13^C-NMR (DMSO-d_6_) δ ppm 156.8, 153.1, 152.0, 147.1, 130.3, 129.4, 129.1, 128.6, 117.2, 87.9, 84.7, 71.2, 62.1, 36.6. UV (MeCN) λ_max_ (log ε) 279 (4.4); MS m/z 344 (MH^+^), MS/MS 344Ψ 228 (C_11_H_10_N_5_O^+^). 

*C8-p-Tolyl-2’-deoxyguanosine* (**3b**): Yield 99%; IR (KBr) cm^-1^ 3600-3260, 3225, 2910, 1690, 1620, 1350, 1080, 1015; ^1^H-NMR (DMSO-d_6_) δ ppm 10.73 (1H, s, NH), 7.53 (2H, d, *J* = 8.40, ArH-3,5), 7.34 (2H, d, *J* = 7.80, ArH-2,6), 6.37 (2H, bs, NH_2_), 6.05 (1H, dd, *J* = 6.6, 7.8 Hz, H-1'), 5.13 (1H, d, *J* = 4.20, 3'-OH), 4.98 (1H, t, *J* = 6.00, 5'-OH), 4.33 (1H, h, *J* = 3.1 Hz, H-3'), 3.78 (1H, m, H-4'), 3.65 (1, p, *J* = 5.4 Hz, H-5"), 3.54 (1H, *J* = 5.4 Hz, H-5'), 3.16 (1H, p, *J* = 6.9 Hz, H-2"), 2.38 (3H, s, Ar-CH_3_), 2.00 (1H, ddd, *J* = 2.4, 6.6, 9 Hz, H-2'); ^13^C-NMR (DMSO-d_6_) δ ppm 156.6, 152.9, 151.9, 147.2, 139.1, 129.2, 129.0, 127.5, 117.1, 87.9, 84.6, 71.2, 62.1, 36.5, 20.9; UV (MeCN) λ_max_ (log ε) 282 (4.5); MS: m/z 358 (MH^+^), MS/MS 358Ψ242 (C_12_H_12_N_5_O^+^). 

*C8-(4-Carboxyphenyl)-2′-deoxyguanosine* (**3c**): Yield 83%; ^1^H-NMR (DMSO-d_6_): δ ppm 10.82 (1H, s, NH), 8.07 (2H, d, *J* = 7.8 Hz, phenyl), 7.80 (2H, d, *J* = 8.4 Hz, phenyl), 6.48 (2H, s, NH_2_), 6.10 (1H, t, *J* = 7.2 Hz, H-1′), 5.14 (1H, brs, 3′-OH), 4.96 (1H, brs, 5′-OH), 4.34 (1H, m, H-3′), 3.80 (1H, m, H-4′), 3.66 and 3.55 (2H, m, H-5′/5′′), 3.11 (1H, m, H-2′′) and 2.05 (1H, ddd, *J* = 3.0, 6.6, 13.2 Hz, H-2’); ^13^C-NMR (DMSO-d_6_): δ ppm 166.9, 156.6, 153.2, 152.3, 146.1, 134.3, 131.4, 129.5, 129.2, 117.4, 87.9, 84.5, 71.0, 61.9, and 36.7. UV: ε^227^ = 13,745 cm^-1^M^-1^ and ε^285^ = 18,054 cm^-1^M^-1^. MS: m/z for MW 387.35, calculated MH^+ ^388.35, found 388, 410 (M+Na)^+^, 426 (M+K)^+^, 775 (2M+H)^+^,and 797 (2M+Na)^+^.

*C8-(4-(TBS-O-methyl)phenyl)-2′-deoxyguanosine* (**3d**): Yield 72%; The TBS protected boronic acid was prepared as described by Zheng, *et al*. [13]; ^1^H-NMR (DMSO-d_6_): δ ppm 10.78 (1H, s, NH), 7.62 (2H, d, *J* = 8.4 Hz, phenyl), 7.46 (2H, d, *J* = 8.4 Hz, phenyl), 6.39 (2H, s, NH_2_), 6.06 (1H, t, *J* = 7.5 Hz, H-1′), 5.12 (1H, d, *J* = 3.6 Hz, 3′-OH), 4.96 (1H, d, *J*=4.2 Hz, 5′-OH), 4.80 (2H, s, CH_2_), 4.34 (1H, m, H-3′), 3.79 (1H, m, H-4′), 3.65 and 3.55 (2H, m, H-5′/5′′), 3.16 (1H, m, H-2′′), 2.02 (1H, m, H-2′), 0.93 (9H, s, t-butyl), and 0.11 (6H, s, dimethylsilyl); ^13^C-NMR (DMSO-d_6_): δ ppm 156.7, 153.0, 151.9, 147.0, 142.5, 129.0, 128.9, 126.0, 117.1, 87.9, 84.7, 71.2, 63.9, 62.1, 36.6, 25.8, 18.0, and -5.3. UV: ε^281^ = 15,285 cm^-1^M^-1^. MS: m/z for MW 487.63, calculated MH^+ ^488.63, found 488, 510 (M+Na)^+^, 526 (M+K)^+^, 975 (2M+H)^+^, and 997 (2M+Na)^+^.

*C8-(4-Methoxymethylphenyl)-2′-deoxyguanosine* (**3e**): Yield 67%; ^1^H-NMR (DMSO-d_6_): δ ppm 10.76 (1H, s, NH), 7.63 (2H, d, *J* = 8.4 Hz, phenyl), 7.47 (2H, d, *J* = 7.8 Hz, phenyl), 6.39 (2H, s, NH_2_), 6.07 (1H, dt, *J* = 1.8, 7.5 Hz, H-1′), 5.12 (1H, d, *J* = 4.2 Hz, 3′-OH), 4.97 (1H, t, *J* = 5.7 Hz, 5′-OH), 4.49 (2H, s, CH_2_), 4.33 (1H, m, H-3′), 3.79 (1H, m, H-4′), 3.65 and 3.54 (2H, m, H-5′/5′′), 3.34 (3H, s, OCH_3_), 3.15 (1H, m, H-2′′) and 2.02 (1H, m, H-2′); ^13^C-NMR (DMSO-d_6_): δ ppm 156.6, 153.0, 152.0, 146.9, 139.7, 129.4, 129.1, 127.5, 117.1, 87.8, 84.6, 73.1, 71.2, 62.1, 57.7, and 36.6. UV: ε^221^ = 9,377 cm^-1^M^-1^ and ε^282^ = 15,552 cm^-1^M^-1^. MS: m/z for MW 387.40, calculated MH^+ ^388.40, found 411 (M+Na)^+^ and 798 (2M+Na)^+^.

*C8-Phenyl-2'-deoxyadenosine* (**8a**): Yield 85%; IR (KBr) cm^-1^ 3500-3000, 2935, 1700-1550, 1480, 1450, 1340, 1300, 1250, 1135, 1100, 1060; ^1^H-NMR (DMSO-d_6_) δ ppm 8.15 (1H, s, H-2), 7.72 (2H, m, ArH-3,5), 7.60 (3H, m, ArH-2,4,6), 7.44 (2H, bs, NH_2_), 6.16 (1H, dd, *J*=6.6, 8.4 Hz, H-1'), 5.55 (1H, bs, 5'-OH), 5.23 (1H, bs, 3'-OH), 4.46 (1H, d, *J* = 4.8Hz, H-3'), 3.88 (1H, h, *J* =2.2 Hz, H-4'), 3.70 (1H, dd, *J* =4.2, 12, H-5"), 3.54 (1H, bd, *J* =12, H-5'), 3.31 (1H, m, H-2"), 2.15 (1H, ddd, *J*=1.8, 6.6, 13.2 H-2'); ^13^C NMR (DMSO-d_6_) δ ppm 156.1, 152.0, 150.4, 149.9, 130.1, 129.7, 129.5, 128.8, 119.2, 88.4, 85.7, 71.5, 62.3, 37.2; UV (MeCN) λ_max_ (log e) 276 (4.12); MS: m/z 328 (MH^+^), MS/MS 328Ψ212 (C_11_H_10_N_5_^+^). 

*C8-p-Tolyl-2'-deoxyadenosine* (**8b**): Yield 80%; IR (KBr) cm^-1^ 3700-2990, 2940, 1655, 1605, 1480, 1335, 1305, 1095; ^1^H NMR (DMSO-d_6_) δ ppm 8.14 (1H, s, H-2), 7.60 (2H, d, *J* = 8.4 Hz, ArH-3,5), 7.40 (2H, d, *J* =8.4 Hz, ArH-2,6), 6.14 (1H, dd, *J* = 6.0, 8.4 Hz, H-1'), 5.57 (1H, bs, 5'-OH), 5.22 (1H, bs, 3'-OH), 4.46 (1H, m, H-3'), 3.87 (1H, h, *J* = 2.2 Hz, H-4'), 3.70 (1H, dd, *J* = 2.1, 12 Hz, H-5"), 3.54 (1H, bm, H-5'), 3.30 (1H, m, H-2"), 2.41 (3H, s Ar-CH_3_), 2.14 (1H, ddd, *J* = 1.8, 6, 12.6 Hz, H-2'); ^13^C-NMR (DMSO-d_6_) δ ppm 156.1, 151.8, 150.6, 149.9, 139.9, 126.8, 129.3, 129.3, 119.1, 88.4, 85.7, 71.5, 62.4, 37.2, 21.0; UV (MeCN) λ_max_ (log ε) 279 (4.28); MS: m/z 342 (MH^+^), MS/MS 342Ψ226 (C_12_H_12_N_5_^+^). 

### General Procedure for Dimethylformamidine Protection

The arylpurine **3** (0.35 g, 1.02 mmol) was co-evaporated with dry pyridine (3 × 2 mL) dissolved in MeOH (7 mL) and *N,N*-dimethylformamidine dimethyl acetal (625 uL, 5.0 mmol) then added (ratio **3 **(mmol):MeOH (mL):acetal (mmol) = 1:7:5). The reaction mixture was stirred at room temperature for 48 hours and then concentrated *in vacuo* to give **4**.

*N2-(N,N-Dimethylformamidine)-C8-phenyl-2'-deoxyguanosine* (**4a**): Yield 92%; IR (KBr) cm^-1^ 3600-3000, 2930, 1680, 1645, 1430, 1345, 1115; ^1^H-NMR (DMSO-d_6_) δ ppm 11.43 (1H, bs, NH), 8.51 (1H, s, CHN), 7.66 (2H, dd, J=1.0, 7.8 Hz, ArH-3,5), 7.55 (3H, m, ArH-2,4,6), 6.11 (1H, t, *J* = 6.6 Hz, H-1'), 5.21 (1H, d, *J* = 4.80 Hz, 3'-OH), 4.89 (1H, t, *J* = 4.8 Hz, 5-'OH), 4.43 (1H, q, *J* = 4.0 Hz, H-3'), 3.81 (1H, q, *J* = 3.0 Hz, H-4'), 3.67 (1H, m, H-5"), 3.57 (1H, m, H-5'), 3.22 (1H, h, *J* = 7.2 Hz, H-2"), 3.16 (3H, s, CH_3_N), 3.05 (3H, s, CH_3_N), 2.08 (1H, ddd, *J* = 2.4, 6.6, 13.2 Hz, H-2'); ^13^C-NMR (DMSO-d_6_) δ ppm 158.1, 157.5, 156.8, 152.1, 150.3, 130.2, 129.5, 129.1, 128.7, 120.2, 87.7, 84.8, 71.0, 62.0, 40.8, 37.0, 34.6; UV (MeCN) λ_max_ (log ε) 317 (4.49) MS m/z 399 (MH^+^), MS/MS 399Ψ283 (C_14_H_15_N_6_O^+^). 

*N2-(N,N-Dimethylformamide)-C8-p-tolyl-2'-deoxyguanosine* (**4b**): Yield 89%; IR (KBr) cm^-1 ^3600-2700, 1675, 1630, 1570-1500, 1430, 1350, 1290; ^1^H-NMR (DMSO-d_6_) δ ppm 11.42 (1H, s, NH), 8.50 (1H, s, HCN), 7.54 (2H, d, *J* = 7.2 Hz, ArH-3,5), 7.36 (2H, d, *J* = 7.2 Hz, ArH-2,6), 6.09 (1H, m, H-1'), 5.23 (1H, m, 3'-OH), 4.91 (1H, m, 5'-OH), 4.43 (1H, m, H-3'), 3.81 (1H, m, H-4'), 3.66 (1H, m, H-5"), 3.57 (1H, m, H-5'), 3.22 (1H, m, H-2"), 3.15 (3H, s, CH_3_N), 3.05 (3H, s, CH_3_N), 2.39 (3H, s, Ar-CH_3_), 2.06 (1H, m, H-2'); ^13^C-NMR (DMSO-d_6_) δ ppm 158.1,157.6, 156.8, 150.6, 148.2, 139.2, 129.2, 129.0, 127.3, 120.1, 87.7, 84.9, 71.0, 62.0, 40.8, 37.0, 34.6, 20.9; UV (MeCN) λ_max_ (log ε) 316 (4.29). MS: m/z 413 (MH^+^), MS/MS 413Ψ297 (C_15_H_17_N_6_O^+^). 

*N2-(N,N-Dimethylformamidine)-C8-(4-carboxyphenyl)-2′-deoxyguanosine* (**4c**): Yield 96%; ^1^H-NMR (DMSO-d_6_): δ ppm 8.53 (1H, s, HC=N), 8.02 (2H, d, *J* = 8.4 Hz, phenyl), 7.64 (2H, d, *J* = 8.4 Hz, phenyl), 6.14 (1H, t, *J* = 7.2 Hz, H-1′), 4.45 (1H, m, H-3′), 3.82 (1H, m, H-4′), 3.68 and 3.58 (2H, m, H-5′/5′′), 3.18 (1H, m, H-2′′), 3.16 and 3.05 (3H each, s, N(CH_3_)_2_), and 2.10 (1H, ddd, *J* = 3.0, 6.6, 13.2 Hz, H-2′); ^13^C-NMR (DMSO-d_6_): δ ppm 169.2, 158.1, 156.8, 150.8, 148.0, 138.7, 131.2, 129.1, 128.5, 120.2, 87.7, 84.7, 71.0, 61.9, 40.8, 37.1, 34.6, and 34.1. UV: ε^231^ = 17,153 cm^-1^M^-1^ and ε^318^ = 20,164 cm^-1^M^-1^; MS: m/z for MW 442.43, calculated MH^+ ^443.43, found 443, 465 (M+Na)^+^, 488 (M+2Na)^+^, 885 (2M+H)^+^, and 907 (2M+Na)^+^.

*N2-(N,N-Dimethylformamidine)-C8-(4-(TBS-O-methyl)phenyl)-2′-deoxyguanosine* (**4d**): Yield 77%; ^1^H-NMR (DMSO-d_6_): δ ppm 11.42 (1H, s, NH), 8.51 (1H, s, HC=N), 7.63 (2H, d, *J* = 7.8 Hz, phenyl), 7.48 (2H, d, *J* = 7.8 Hz, phenyl), 6.11 (1H, t, *J* = 7.2 Hz, H-1′), 5.20 (1H, d, *J* = 2.4 Hz, 3′-OH), 4.88 (1H, m, 5′-OH), 4.81 (2H, s, CH_2_), 4.44 (1H, m, H-3′), 3.82 (1H, m, H-4′), 3.67 and 3.57 (2H, m, H-5′/5′′), 3.22 (1H, m, H-2′′), 3.16 and 3.05 (3H each, s, N(CH_3_)_2_), 2.08 (1H, m, H-2′), 0.93 (9H, s, t-butyl), and 0.12 (6H, s, dimethylsilyl); ^13^C-NMR (DMSO-d_6_): δ ppm 158.1, 157.5, 156.8, 150.6, 148.0, 142.7, 129.0, 128.7, 126.0, 120.2, 87.7, 84.8, 71.0, 63.9, 62.0, 40.8, 37.0, 34.6, 25.8, 18.0, and -5.4. UV: ε^229^ = 21,603 cm^-1^M^-1^ and ε^313^ = 28,129 cm^-1^M^-1^; MS: m/z for MW 542.71, calculated MH^+ ^543.71, found 543, 565 (M+Na)^+^, 1085 (2M)^+^, and 1107 (2M+Na)^+^.

*N2-(N,N-Dimethylformamidine)-C8-(4-methoxymethylphenyl)-2′-deoxyguanosine* (**4e**): Yield 90%; ^1^H-NMR (DMSO-d_6_): δ ppm 11.45 (1H, s, NH), 8.51 (1H, s, HC=N), 7.64 (2H, d, *J* = 8.4 Hz, phenyl), 7.49 (2H, d, *J* = 7.8 Hz, phenyl), 6.12 (1H, t, *J* = 7.2 Hz, H-1′), 5.21 (1H, d, *J* = 4.2 Hz, 3′-OH), 4.88 (1H, dt, *J* = 1.8, 6.0 Hz, 5′-OH), 4.50 (2H, s, CH_2_), 4.44 (1H, m, H-3′), 3.82 (1H, m, H-4′), 3.68 and 3.57 (2H, m, H-5′/5′′), 3.34 (3H, s, OCH_3_ ), 3.22 (1H, m, H-2′′), 3.16 and 3.05 (3H each, s, N(CH_3_)_2_), and 2.08 (1H, m, H-2′); ^13^C-NMR (DMSO-d_6_): δ ppm 158.1, 157.5, 156.8, 150.7, 147.9, 139.9, 129.2, 129.1, 127.6, 120.2, 87.7, 84.8, 73.1, 71.0, 62.0, 57.7, 40.8, 37.0, and 34.6. UV: ε^229^ = 14,602 cm^-1^M^-1^ and ε^314^ = 20,834 cm^-1^M^-1^; MS: m/z for MW 442.47, calculated MH^+ ^443.47, found 443, 466 (M+Na)^+^, and 908 (2M+Na)^+^.

*N6-(N,N-Dimethylformamide)-C8-phenyl-2'-deoxyadenosine* (**9a**): Yield 64%; IR (KBr) cm^-1^ 3650-3000, 2935, 1640, 1570, 1450, 1420, 1340, 1100; ^1^H-NMR (DMSO-d_6_) δ ppm 8.93 (1H, s, HCN), 8.43 (1H, s, H-2), 7.76 (2H, m, ArH-3,5), 7.62 (3H, m, ArH-2,4,6), 6.19 (1H, dd, *J* = 6.6, 8.4, H-1'), 5.34 (1H, bs, 5'-OH), 5.27 (1H, bs, 3'-OH), 4.48 (1H, m, H-3'), 3.87 (1H, h, *J* = 2.3 Hz, H-4'), 3.71 (1H, bd, *J* = 11.4 Hz, H-5"), 3.54 (1H, m, H-5'), 3.36 (1H, m, H-2"), 3.20 (3H, s, CH_3_N), 3.14 (3H, s, CH_3_N), 2.17 (1H, ddd, *J* = 2.4, 6.6, 13.2 Hz, H-2'); ^13^C-NMR (DMSO-d_6_) δ ppm 159.2, 157.9, 152.2, 152.1, 151.2, 130.2, 129.7, 129.5, 128.8, 125.7, 88.2, 85.5, 71.3, 62.2, 40.7, 36.9, 34.6; UV (MeCN) λ_max_ (log ε) 322 (4.38), 256 (4.12); MS: m/z 383 (MH^+^), MS/MS 383Ψ267 (C_14_H_15_N_6_^+^). 

*N6-(N,N-Dimethylformamide)-C8-p-tolyl-2'-deoxyadenosine* (**9b**): Yield 97%; IR (KBr) cm^-1^ 3500-3100, 2920, 1630, 1570, 1460, 1420, 1330, 1110, 1090, 1050; ^1^H-NMR (DMSO-d_6_) δ ppm 8.92 (1H, s, HCN), 8.42 (1H, s, H-2), 7.64 (2H, d, *J* = 8.40, ArH-3,5), 7.42 (2H, d, *J* = 8.4, ArH-2,6), 6.18 (1H, t, *J* =7.2 Hz, H-1'), 5.37 (1H, bs, 5'-OH), 5.24 (1H, bs, 3'-OH), 4.48 (1H, m, H-3'), 3.87 (1H, m, H-4'), 3.71 (1H, bd, *J* = 12 Hz, H-5"), 3.54 (1H, bd, *J* = 12 Hz, H-5'), 3.35 (1H, m, H-2"), 3.20 (3H, s, CH_3_N), 3.13 (3H, s, CH_3_N), 2.42 (3H, s, Ar-CH_3_), 2.15 (1H, bdd, *J* = 6, 13.8 Hz, H-2'); ^13^C-NMR (DMSO-d_6_) δ ppm 159.1, 157.8, 152.3, 152.0, 151.1, 126.8, 140.1, 129.3, 129.4, 125.7, 88.2, 85.5, 71.4, 62.2, 40.7, 36.9, 34.6, 21.0; UV (MeCN) λ_max_ (log ε) 258 (4.31), 323 (4.56); MS: m/z 397 (MH^+^), MS/MS 397Ψ281 (C_15_H_17_N_6_^+^). 

### General Procedure for Dimethoxytrityl Protection

Compound **4** (0.63 mmol) was dried *in vacuo* (P_2_O_5_), dissolved in pyridine (PYR) (5 mL), TEA (0.11 mL) added and then 4,4'-dimethoxytritylchloride (DMTrCl) (0.314 mg, 0.93 mmol) (ratio **4** (mmol):PYR (mL):TEA (mL):DMTrCl (mmol) = 47:1:6:8.9). The reaction mixture stirred for 2.5 hours at room temperature, MeOH (3 mL) added to quench the reaction, the solvent was removed *in vacuo* and residual purified (alumina, MeOH:CH_2_Cl_2_, 0-10%). 

*5'-O-(4,4'-Dimethoxytrityl)-N2-(N,N-dimethylformamidine)-C8-phenyl-2'-deoxyguanosine* (**5a**): Yield 75%; IR (KBr) cm^-1^ 3600-3150, 3065, 2940, 1685, 1630, 1570-1490, 1440, 1340, 1250, 1175, 1110, 1030. ^1^H-NMR (acetone-d_6_) δ ppm 8.32 (1H, s, HCN), 7.79 (2H, m, ArH-3,5), 7.51 (3H, m, 3H, ArH-2,4,6), 7.37 (2H, d, *J* = 7.8 Hz, ArH-3',5'), 7.249 and 7.238 (4H, d, *J* = 9.0 Hz, ArH-3",5"), 7.19 (3H, m, ArH-2',4',6'), 6.752 and 6.718 (4H, d, J = 9.0 Hz, ArH-2",6"), 6.18 (1H, dd, J = 5.4, 7.8 Hz, H-1'), 4.68 (1H, dt, *J* = 5.4, 7.2 Hz, H-3'), 3.98 (1H, m, H-4'), 3.73 (3H, s, CH_3_O), 3.72 (3H, s, CH_3_O), 3.40 (1H, dd, *J* = 7.2, 9.9 Hz, H-5"), 3.34 (1H, m, H-2"), 3.20 (1H, dd, *J* = 3.6, 9.9 Hz, H-5'), 3.03 (3H, s, CH_3_N), 2.95 (3H, s, CH_3_N), 2.17 (1H, m, H-2'); ^13^C-NMR (acetone-d_6_) δ ppm 159.6, 159.0, 158.7, 157.4, 152.1, 150.3, 146.4, 137.3, 137.0, 131.7, 131.1, 130.6, 130.5, 129.7, 129.1, 128.8, 127.8, 121.6, 114.0, 87.3, 85.8, 72.8, 65.5, 56.0, 55.9, 41.8, 38.4, 35.4; UV (MeCN) λ_max_ (log ε) 320 (4.44); MS: m/z 701 (MH^+^), MS/MS 701Ψ303 (C_21_H_19_O_2_^+^), MS/MS 701Ψ283 (C_14_H_15_N_6_O^+^). 

*5'-O-(4,4'-Dimethoxytrityl)-N2-(N,N-dimethylformamidine)-C8-p-tolyl-2'-deoxyguanosine* (**5b**): Yield: 27%; IR (KBr) cm^-1^ 2940, 1690, 1635, 1510, 1250, 1175, 1030; ^1^H-NMR (MeCN-d_3_) δ ppm 8.31 (1H, s, HCN), 7.63 (2H, d, *J* = 8.0 Hz, ArH-3,5), 7.36 (2H, d, *J* = 7.9 Hz, DMTrH-3',5'), 7.29 (2H, d*, J* = 8.0 Hz, ArH-2,6), 7.24 and 7.23 (4H, m, DMTrH-3’,3",5’,5"), 7.18 (2H, m, DMTrH-2,6), 7.16 (1H, m, DMTrH-4'), 6.74 and 6.71 (4H, d, *J* = 6.72 Hz, DMTrH-2’,2",6’,6"), 6.17 (1H, dd, *J* = 5.4, 8.0 Hz, H-1'), 4.68 (1H, q, *J* = 5.7 Hz, H-3'), 3.98 (1H, ddd, *J* = 3, 5, 7.5 Hz, H-4'), 3.71 (3H, s, CH_3_O), 3.70 (3H, s, CH_3_O), 3.39 (1H, dd, *J* = 7.5, 9.9 Hz, H-5"), 3.33 (1H, ddd, *J* = 5.0, 7.1, 13.3 Hz, H-2''), 3.20 (1H, dd, *J* = 3.0, 9.9 Hz, H-5'), 3.00 (3H, s, CH_3_N), 2.93 (3H, s, CH_3_N), 2.40 (3H, s, Ar-CH_3_), 2.17 (1H, ddd, *J* =5.7, 8.2, 13.3 Hz, H-2'); ^13^C-NMR (MeCN-d_3_) δ ppm 159.5, 158.9,158.8, 157.2, 152.0, 150.4, 146.5, 140.9, 137.2, 137.0, 130.4, 131.0, 130.0, 129.0, 128.7, 128.0, 127.8, 121.2, 114.0, 87.2, 85.7, 72.8, 65.5, 56.0, 55.9, 41.8, 38.3, 35.3, 21.5; UV (MeCN) λ_max_ (log ε) 322 (4.41), 231 (4.62); MS: m/z 715 (MH^+^), MS/MS 715Ψ303 (C_21_H_19_O_2_^+^), MS/MS 715Ψ297 (C_15_H_17_N_6_O^+^). 

*5′-O-(DMTr)-N2-(N,N-dimethylformamidine)-C8-(4-carboxyphenyl)-2′-deoxyguanosine* (**5c**): Yield 31%; ^1^H-NMR (DMSO-d_6_): δ ppm 8.32 (1H, s, HC=N), 8.05 (2H, d, *J* = 7.8, phenyl), 7.83 (2H, d, *J* = 8.4, phenyl), 7.32 (2H, d, *J* = 8.2 Hz, DMTr-H), 7.17-7.2 (3H, m, DMTrH-3,4,5), 7.19 (2H, d, *J* = 7.8 Hz, DMTrH-3’,5’), 7.18 (2H, d, *J* = 7.8 Hz, DMTrH-3”,5”), 6.77 (2H, d, *J* = 7.8 Hz, DMTrH-2”,6”), 6.73 (2H, d, *J* = 7.8 Hz, DMTrH-2’,6’), 6.20 (1H, dd, *J* = 4.8, 7.8, H-1′), 4.57 (1H, q, *J* = 5.9 Hz, H-3′), 3.93 (1H, m, H-4′), 3.71 and 3.70 (3H each, s, OCH_3_), 3.30 (1H, dd, *J* = 7.2,10 Hz, H-5”), 3.21 (1H, ddd, *J* = 5.4, 7.2, 13.4 Hz, H-2′′), 3.15 (1H, dd, *J* = 2.8,10 Hz, H-5′), 3.03 and 2.98 (3H each, s, N(CH_3_)_2_), and 2.20 (1H, ddd, J = 6.0,8.0,13.4 Hz, H-2′); ^13^C-NMR (DMSO-d_6_): δ ppm 157.9, 157.9, 157.6, 157.5, 156.4, 150.5, 147.5, 144.9, 135.7, 135.6, 129.6, 129.4, 129.3, 129.0, 127.6, 127.6, 126.5, 120.3, 112.9, 112.9, 85.7, 85.2, 84.1, 70.8, 64.0, 54.9, 54.9, 40.7, 37.4, 34.6, and 34.2; UV: (MeCN) λ_max_ (log ε) 233 (4.63) and 322 (4.44); MS: m/z for MW 744.80, calculated MH^+ ^745.80, found 745.767 (M+Na)^+^, and 1511.0 (2M+Na)^+^.

*5′-O-(DMTr)-N2-(N,N-dimethylformamidine)-C8-(4-(TBS-O-methyl)phenyl)-2′-deoxyguanosine* (**5d**): Yield 41%; ^1^H-NMR (DMSO-d_6_): δ ppm 11.41 (1H, s, NH), 8.31 (1H, s, HC=N), 7.74 and 7.46 (4H, d, *J* = 8.4, ArH-2,6 and ArH-3,5), 7.33 (2H, d, *J* = 8.4 Hz, DMTrH-2,6), 7.24 (3H, m, DMTrH-3,4,5), 7.21 (2H, d, *J* = 8.5 Hz, DMTrH-3’,5’), 7.18 (2H, d, *J* = 8.5 Hz, DMTrH-3”,5”), 6.77 (2H, d, *J* = 8.5 Hz, DMTrH-2’,6’), 6.73 (2H, d, *J* = 8.5 Hz, DMTrH-2”,6”), 6.17 (1H, dd, *J* = 5.4,7.8 Hz, H-1′), 5.28 (1H, d, *J* = 5.6 Hz, 3′-OH), 4.81 (2H, s, CH_2_), 4.57 (1H, p, *J* = 5.1 Hz, H-3′), 3.93 (1H, m, H-4′), 3.71 and 3.70 (3H each, s, OCH_3_), 3.32 (1H, dd, *J* = 6.3, 10.2 Hz, H-5’or 5′′), 3.21 (1H, p, *J* = 6.3 Hz, H-2′′), 3.15 (2H, dd, *J* = 2.8, 10.2 Hz, H-5′ or 5′′), 3.03 and 2.98 (3H each, s, N(CH_3_)_2_), 2.18 (1H, ddd, *J* = 5.8,8,13.7, H-2′), 0.94 (9H, s, t-butyl), and 0.12 (6H, s, dimethylsilyl); ^13^C-NMR (DMSO-d_6_): δ ppm 157.9, 157.9, 157.5, 156.3, 150.4, 148.2, 144.9, 142.6, 135.7, 135.6, 129.6, 129.4, 129.1, 128.9, 127.6, 127.6, 126.5, 126.0, 120.0, 113.1, 112.9, 112.9, 85.7, 85.2, 84.0, 70.9, 64.0, 63.9, 54.9, 54.9, 40.7, 37.4, 34.6, 25.8, 18.0, and -5.4; UV: (MeCN) λ_max_ (log ε) 231 (4.60) 316 (4.36); MS: m/z for MW 845.08, calculated MH^+ ^846.08, found 845, 867 (M+Na)^+^, and 1712 (2M+Na)^+^.

*5′-O-(DMTr)-N2-(N,N-dimethylformamidine)-C8-(4-methoxymethylphenyl)-2′-deoxyguanosine* (**5e**): Yield 55%; ^1^H-NMR (DMSO-d_6_): δ ppm 11.42 (1H, s, NH), 8.31 (1H, s, HC=N), 7.74 (2H, d, *J* = 8.4 Hz, ArH-2,6 or 3,5), 7.46 (2H, d, *J* = 8.4, ArH-2,6 or 3,5), 7.32 (2H, d, *J* =8.9 Hz, DMTrH-2,6), 7.19 (2H, d, *J* = 8.7 Hz, DMTrH-3’,5’), 7.18 (2H, d, *J* = 8.7 Hz, DMTrH-3”,5”), 7.14-7.18 (3H, m, ArH-3,4 5), 6.77 (2H, d, *J* = 8.7 Hz, DMTrH-2”,6”), 6.73 (2H, d, *J* = 8.7 Hz, DMTrH-2’,6’), 6.17 (1H, dd, *J* = 3.0, 6.6 Hz, H-1′), 5.28 (1H, d, *J* = 4.9 Hz, 3′-OH), 4.57 (1H, p, 5.5 Hz, H-3′), 4.50 (2H, s,CH_2_), 3.92 (1H, m, H-4′), 3.71 and 3.70 (3H each, s, OCH_3_), 3.35 (3H, s, CH_3_), 3.30 (1H, dd, *J* = 7,9.9 Hz, H-5′ or 5′′), 3.20 (1H, ddd, *J* = 5,6.8,13 Hz, H-2′′), 3.15 (1H, dd, *J* = 3, 9.9 Hz, H-5′ or 5′′), 3.03 and 2.98 (3H each, s, N(CH_3_)_2_), and 2.18 (1H, ddd, 5.7, 8, 13.5 Hz, H-2′); ^13^C-NMR (DMSO-d_6_): δ ppm 157.9, 157.9, 157.5, 156.3, 150.4, 148.1, 144.9, 139.8, 135.7, 135.6, 129.6, 129.4, 129.1, 127.6, 127.6, 127.5, 126.5, 120.1, 112.9, 112.9, 85.7, 85.2, 84.0, 73.1, 70.9, 64.0, 57.7, 54.9, 54.9, 40.7, 37.4, and 34.6; UV: (MeCN) λ_max_ (log ε) 231 (4.54) and 319 (4.37); MS: m/z for MW 744.85, calculated MH^+ ^745.85, found 746, 768 (M+Na)^+^, 784 (M+K)^+^, and 1512 (2M+Na)^+^.

*5'-O-(4,4'-Dimethoxytrityl)-N2-(N,N-dimethylformamidine)-C8-phenyl-2'-deoxyadenosine* (**10a**): Yield 58%; IR (KBr) cm^-1^ 3500-3130, 2940, 2600, 2500, 1670-1530, 1505, 1440, 1420, 1330, 1250, 1170, 1110, 1040; ^1^H-NMR (MeCN-d_3_) δ ppm 8.87 (1H, s, HCN), 8.20 (1H, s, H-2), 7.83 (2H, d, *J* = 7.3 Hz, ArH-3,5), 7.55 (3H, m, ArH-2,6), 7.38 (2H, d, *J* = 7Hz DMTrH-2”,6”), 7.25 and 7.24 (4H, d, *J* = 8.8 Hz, DMTrH-3’,3",5’,5"), 7.19 (3H, m, DMTrH-3”,4”,5”), 6.85 and 6.75 (4H, d, *J* = 8.8 Hz, DMTrH-2’,2",6’,6"), 6.21 (1H, t, *J* = 7.0 Hz, H-1'), 4.74 (1H, m, H- 3'), 4.07 (1H, m, H-4'), 3.73 (3H, s, CH_3_O), 3.70 (3H, s, CH_3_O), 3.53 (1H, dd, *J* = 6.9, 10.3 Hz, H-2"), 3.37 (1H, dd, *J* = 4.4, 10.3 Hz, H-5"), 3.28 (1H, dt, *J* = 7, 13.5 Hz, H-5'), 3.16 (3H, s, CH_3_N), 3.13 (3H, s, CH_3_N), 2.17 (1H, ddd, *J* = 4.2, 7.9, 13.5 Hz, H-2'). ^13^C-NMR (MeCN-d_3_) δ ppm 159.6, 159.5, 158.9, 154.2, 153.8, 152.5, 146.4, 137.3, 137.1, 137.0, 131.1, 130.8, 130.1, 129.7, 129.1, 128.7, 127.7, 124.8, 114.0, 87.4, 86.2, 73.1, 65.4, 56.0, 55.9, 41.6, 37.4, 35.3; UV(MeCN) λ_max_ (log ε) 320 (4.25); MS: m/z 685 (MH^+^), MS/MS 685Ψ303 (C_21_H_19_O_2_^+^), MS/MS 685Ψ267 (C_14_H_15_N_6_^+^). 

*5'-O-(4,4'-Dimethoxytrityl)-N2-(N,N-dimethylformamidine)-C8-p-tolyl-2'-deoxyadenosine* (**10b**): Yield 86%; IR (KBr) cm^-1^ 2940, 1610, 1560, 1250, 1180, 1035; ^1^H-NMR (MeCN-d_3_) δ ppm 8.86 (1H, s, HCN), 8.21 (1H, s, H-2), 7.73 (2H, m, ArH-3,5), 7.38 (2H, m, ArH-3',5'), 7.35 (2H, m, ArH-2,6), 7.25 (4H, m, ArH-3",5"), 7.22 (2H, m, ArH-2',6'), 7.19 (1H, m, ArH-4'), 6.74 (4H, m, ArH-2",6"), 6.23 (1H, m, H-1'), 4.74 (1H, m, H- 3'), 4.05 (1H, m, H-4'), 3.71 (3H, s, CH_3_O), 3.70 (3H, s, CH_3_O), 3.52 (1H, m, H-5"), 3.34 (1H, m, H-2"), 3.29 (1H, m, H-5'), 3.14 (3H, s, CH_3_N), 3.13 (3H, s, CH_3_N), 2.41 (3H, s, ArCH_3_), 2.13 (1H, m, H-2'); ^13^C-NMR (MeCN-d_3_) δ ppm 159.7, 158.9, 156.7, 153.0, 152.4, 151.8, 146.4, 140.6, 137.3, 137.1, 131.1, 130.9, 130.4, 129.1, 128.7, 127.7, 127.2, 124.8, 114.0, 87.4, 86.4, 73.1, 65.3, 56.0, 55.9, 41.5, 37.4, 35.3, 21.6; UV (MeCN) λ_max_ (log ε) 234 (4.63); MS: m/z 699 (MH^+^), MS/MS 699Ψ303 (C_21_H_19_O_2_^+^), MS/MS 699Ψ281 (C_15_H_17_N_6_^+^). 

### General Procedure for Phosphoramidite Synthesis

Compound **5** (0.29 mmol) was dried *in vacuo* (P_2_O_5_). CH_2_Cl_2_ (3 mL), TEA (80 μL), and 2-cyanoethyl diisopropylchlorophosphoramidite (CEDClP) (65 μL, 0.29 mmol) were added, the reaction at room temperature for 30 min, a second addition of 2-cyanoethyl diisopropylchlorophosphoramidite (32 μL, 0.14 mmol) made, and stirring at room temperature continued for 30 min (Ratio **5** (mmol):TEA (mmol):CEDClP (mmol):2nd CEDClP (mmol) for all reactions = 2:4:2:1). The reaction solvent was removed *in vacuo*, THF:benzene (1:4, 4 mL) added, the mixture stirred for 10 minutes, and then filtered and the filtrate concentrated *in vacuo*. The residual solid was purified by low pressure alumina chromatography (100% EtOAc).

*3*′*-O-[(2-Cyanoethoxy)(diisopropylamino)phosphino]-5*′*-O-(4,4*′*-dimethoxytrityl)-N2-(N,N-dimethyl-formamidine)-C8-phenyl-2*′*-deoxyguanosine* (**6a**): Yield 44%; IR (KBr) cm^-1^ 2940, 1675, 1630, 1550-1500, 1430, 1350, 1250, 1180, 1120; ^1^H-NMR (acetone-d_6_) δ ppm 8.39 (1H, s, HCN), 7.80 (2H, m, ArH-3,5), 7.54 (3H, m, ArH-2,4,6), 7.35 (2H, m, ArH-3',5'), 7.23 (4H, m, ArH-3",5"), 7.19 (2H, m, ArH-2',6'), 7.17 (1H, m, ArH-4'), 6.74 (4H, m, ArH-2",6"), 6.21 (1H, m, H-1'), 5.04 (1H, m, H- 3'), 4.10 (1H, m, H-4'), 4.04 (2H, m, CH_2_O), 3.75 (3H, s, CH_3_O), 3.73 (3H, s, CH_3_O), 3.68 (1H, m, H-2"), 3.53 (2H, m, CH(CH_3_)_2_). 3.36 (1H, m, H-5"), 3.29 (1H, m, H-5'), 3.05 (3H, s, CH_3_N), 3.01 (3H, s, CH_3_N), 2.72 (2H, m, CH_2_CN), 2.35 (1H, m, H-2'), 1.13 (12H, m, CH(CH_3_)_2_); ^13^C-NMR (acetone-d_6_) δ ppm 159.7, 158.9, 158.6, 157.6, 151.8, 150.2, 146.1, 137.1, 137.0, 136.9, 130.8, 130.7, 130.5, 129.7), 129.0, 128.9, 127.8, 121.6, 118.4, 114.0, 87.0, 85.0, 76.0, 63.7, 61.0, 56.0, 55.9, 47.1, 41.5, 36.9, 35.5, 24.9, 21.0; UV (MeCN) λ_max_ (log ε) 323 (4.26), 285 (4.07); MS: m/z 901 (MH^+^), MS/MS 901Ψ683 (C_40_H_39_N_6_O_5_^+^), MS/MS 901Ψ 619 (C_36_H_45_NO_6_P^+^), MS/MS 901Ψ 303 (C_21_H_19_O_2_^+^), MS/MS 901Ψ283 (C_14_H_15_N_6_O^+^). 

*3*′*-O-[(2-Cyanoethoxy)(diisopropylamino)phosphino]-5*′*-O-(4,4*′*-dimethoxytrityl)-N6-(N,N-dimethyl-formamidine)-C8-p-tolyl-2*′*-deoxyguanosine* (**6b**): Yield 44%; IR (KBr) cm^-1^ 2970, 1700, 1630, 1540, 1510, 1345, 1250, 1170, 1115, 1030; ^1^H-NMR (MeCN-d_3_) δ ppm 8.49 (1H, s, HCN), 7.79 (2H, m, ArH-3,5), 7.46 (2H, m, ArH-2,6), 7.50 (2H, m, ArH-3',5'), 7.32 (4H, m, ArH-3",5"), 7.28 (2H, m, ArH-2',6'), 7.27 (1H, m, ArH-4'), 6.85 (4H, m, ArH-2",6"), 6.31 (1H, m, H-1'), 5.16 (1H, m, H- 3'), 4.21 (1H, m, H-4'), 3.85 (3H, s, CH_3_O), 3.84 (3H, s, CH_3_O), 3.78 (2H, m, CH_2_O), 3.64 (2H, m, CH(CH_3_)_2_). 3.46 (1H, m, H-5"), 3.44 (1H, m, H-5'), 3.35 (1H, m, H-2"), 3.15 (3H, s, CH_3_N), 3.11 (3H, s, CH_3_N), 2.66 (2H, m, CH_2_CN), 2.53 (3H, s, ArCH_3_), 2.44 (1H, m, H-2'), 1.23 (12H, m, CH(CH_3_)_2_); ^13^C-NMR (MeCN-d_3_) δ ppm 159.6, 158.8, 158.1, 157.4, 151.9, 150.9, 146.3, 141.0, 137.2, 137.0, 131.1, 130.4, 130.3, 129.1, 128.8, 128.0, 127.8, 121.6, 119.4, 114.0, 86.9, 85.4, 77.4, 64.7, 59.5, 56.0, 55.9, 44.2, 41.9, 38.1, 35.4, 24.9, 21.1; UV (MeCN) λ_max_ (log ε) 322 (4.44), 285 (4.27); MS: m/z 915 (MH^+^), MS/MS 915Ψ697 (C_41_H_41_N_6_O_5_^+^), MS/MS 915Ψ619 (C_36_H_45_NO_6_P^+^), MS/MS 915Ψ303 (C_21_H_19_O_2_^+^), MS/MS 915Ψ297 (C_15_H_17_N_6_O^+^). 

*3*′*-O-[(2-Cyanoethoxy)(diisopropylamino)phosphino]-5*′*-O-(4,4*′*-dimethoxytrityl)-N6-(N,N-dimethyl-formamidine)-C8-phenyl-2*′*-deoxyadenosine* (**11a**): Yield 90%; IR (KBr) cm^-1^ 2970, 1610, 1560, 1510, 1440, 1415, 1340, 1245, 1175, 1030; ^1^H-NMR (CDCl_3_) δ ppm 8.94 (1H, s, HCN), 8.31 (1H, s, H-2), 7.89 (2H, m, ArH-3,5), 7.51 (3H, m, ArH-2,4,6), 7.43 (2H, m, ArH-3',5'), 7.31 (4H, m, ArH-3",5"), 7.20 (2H, m, ArH-2',6'), 7.17 (1H, m, ArH-4'), 6.75 (4H, m, ArH-2",6"), 6.23 (1H, m, H-1'), 5.06 (1H, m, H- 3'), 4.30 (1H, m, H-4'), 3.78 (3H, s, CH_3_O), 3.76 (3H, s, CH_3_O), 3.74 (2H, m, CH_2_O), 3.68 (1H, m, H-2"), 3.56 (2H, m, CH(CH_3_)_2_), 3.51 (1H, m, H-5"), 3.44 (1H, m, H-5'), 3.26 (3H, s, CH_3_N), 3.20 (3H, s, CH_3_N), 2.54 (2H, m, CH_2_CN), 2.32 (1H, m, H-2'), 1.18 (12H, m, CH(CH_3_)_2_); ^13^CNMR (CDCl_3_) δ ppm 159.3, 158.4, 158.1, 153.5, 152.8, 151.9, 145.0, 136.4, 136.3, 136.2, 130.3, 130.2, 130.1, 128.7, 128.4, 127.8, 126.8, 117.5, 117.0, 113.0, 85.6, 85.3, 74.8, 58.5, 55.5, 55.4, 45.6, 43.5, 41.5, 36.2, 35.5, 24.8, 20.5; UV(MeCN) λ_max_ (log ε) 321 (4.33); MS: m/z 885 (MH^+^), MS/MS 885Ψ667 (C40H39N6O4^+^), MS/MS 885Ψ619 (C_36_H_45_NO_6_P^+^), MS/MS 885Ψ303 (C_21_H_19_O_2_^+^), MS/MS 885Ψ267 (C_14_H_15_N_6_^+^).

*3*′*-O-[(2-Cyanoethoxy)(diisopropylamino)phosphino]-5*′*-O-(4,4*′*-dimethoxytrityl)-N6-(N,N-dimethyl-formamidine)-C8-p-tolyl-2*′*-deoxyadenosine* (**11b**): Yield 17%; IR (KBr) cm^-1^ 2975, 1570, 1510, 1420, 1340, 1250, 1175, 1030; ^1^H-NMR (MeCN-d_3_) δ ppm 8.87 (1H, s, HCN), 8.25 (1H, s, H-2), 7.74 (2H, m, ArH-3,5), 7.38 (2H, m, ArH-3',5'), 7.36 (2H, m, ArH-2,6), 7.24 (4H, m, ArH-3",5"), 7.20 (2H, m, ArH-2',6'), 7.18 (1H, m, ArH-4'), 6.75 (4H, m, ArH-2",6"), 6.25 (1H, m, H-1'), 5.12 (1H, m, H- 3'), 4.17 (1H, m, H-4'), 4.07 (2H, m, CH_2_O), 3.73 (3H, s, CH_3_O), 3.71 (3H, s, CH_3_O), 3.68 (1H, m, H-2"), 3.55 (2H, m, CH(CH_3_)_2_), 3.38 (1H, m, H-5"), 3.31 (1H, m, H-5'), 3.18 (3H, s, CH_3_N), 3.15 (3H, s, CH_3_N), 2.53 (2H, m, CH_2_CN, 2.48 (1H, m, H-2'), 2.43 (3H, s, ArCH_3_), 1.16 (12H, m, CH(CH_3_)_2_); ^13^C- NMR (MeCN-d_3_) δ ppm 159.6, 158.9, 154.2, 153.6, 152.5, 152.2, 146.3, 141.6, 137.4, 137.1, 131.0, 130.7, 130.4, 129.0, 128.7, 128.5, 127.7, 127.0, 119.5, 113.9, 86.4, 85.9, 74.5, 64.9, 59.7, 56.0, 55.9, 44.0, 41.5, 37.0, 35.2, 25.0, 21.6, 21.1; UV(MeCN) λ_max_ (log ε) 321 (4.47), 235 (4.53); MS: m/z 889 (MH^+^), MS/MS 899Ψ681 (C_41_H_41_N_6_O_4_^+^), MS/MS 899Ψ619 (C_36_H_45_NO_6_P^+^), MS/MS 899Ψ303 (C_21_H_19_O_2_^+^), MS/MS 899Ψ281 (C_15_H_17_N_6_^+^).

## 4. Conclusions

We have developed a general approach to the synthesis of C8-arylpurine phosphoramidites. The method is very simple and minimizes the use of protection/deprotection steps. While here we have only coupled phenyl and substituted phenyl groups to either 2′-deoxyguanosine or 2′-deoxyadenosine, a wide range or aromatic groups with various substituents should work as well. The principle difficulty in preparing the C8-arylpurines is that the glycosidic bond is very easily cleaved by acid. Suitable selection of the amino protecting groups has attenuated this problem. Nevertheless, they are still acid sensitive and, if necessary, must be purified on alumina. The reactions are sufficiently clean such that chromatographic purification is not generally necessary, except for the preparation of analytically pure samples. The phosphoramidites have been successfully used for the preparation of oligonucleotices and though the acid sensitivity of the C8-arylguanines and C8-aryladenines reduces the overall coupling efficiency (89% for 10-mers) from that typically observed (98-99%), sufficient quantities of modified oligonucleotides can be easily prepared for, for example, NMR studies.
